# Artificial Intelligence and COVID-19: Deep Learning Approaches for Diagnosis and Treatment

**DOI:** 10.1109/ACCESS.2020.3001973

**Published:** 2020-06-12

**Authors:** Mohammad Behdad Jamshidi, Ali Lalbakhsh, Jakub Talla, Zdeněk Peroutka, Farimah Hadjilooei, Pedram Lalbakhsh, Morteza Jamshidi, Luigi La Spada, Mirhamed Mirmozafari, Mojgan Dehghani, Asal Sabet, Saeed Roshani, Sobhan Roshani, Nima Bayat-Makou, Bahare Mohamadzade, Zahra Malek, Alireza Jamshidi, Sarah Kiani, Hamed Hashemi-Dezaki, Wahab Mohyuddin

**Affiliations:** 1 Department of Electromechanical Engineering and Power Electronics (KEV)University of West Bohemia in Pilsen301 00PilsenCzech Republic; 2 School of EngineeringMacquarie University 7788 SydneyNSW2109Australia; 3 Regional Innovation Centre for Electrical engineering (RICE)University of West Bohemia in Pilsen301 00PilsenCzech Republic; 4 Department of Radiation OncologyCancer Institute, Tehran University of Medical Sciences 48439 Tehran1416753955Iran; 5 Department of English Language and LiteratureRazi University 48494 Kermanshah6714414971Iran; 6 Young Researchers and Elite Club, Kermanshah BranchIslamic Azad University 68106 Kermanshah1477893855Iran; 7 School of Engineering and the Built EnvironmentEdinburgh Napier University 3121 EdinburghEH11 4DYU.K.; 8 Department of Electrical and Computer EngineeringUniversity of Wisconsin–Madison 5228 MadisonWI53706USA; 9 Physics and Astronomy DepartmentLouisiana State University 5779 Baton RougeLA70803USA; 10 Irma Lerma Rangel College of PharmacyTexas A&M UniversityKingsvilleTX78363USA; 11 Department of Electrical EngineeringKermanshah Branch, Islamic Azad University 68106 Kermanshah1477893855Iran; 12 The Edward S. Rogers, Sr. Department of Electrical and Computer EngineeringUniversity of Toronto 7938 TorontoON M5SCanada; 13 Medical Sciences Research Center, Faculty of Medicine, Tehran Medical Sciences BranchIslamic Azad University 68106 Tehran1477893855Iran; 14 Dentistry SchoolBabol University of Medical SciencesBabol4717647745Iran; 15 Medical Biology Research CenterHealth Technology Institute, Kermanshah University of Medical Sciences 48464 Kermanshah6715847141Iran; 16 Research Institute for Microwave and Millimeter-Wave Studies, National University of Sciences and Technology 66959 Islamabad24090Pakistan

**Keywords:** Artificial intelligence, big data, bioinformatics, biomedical informatics, COVID-19, deep learning, diagnosis, machine learning, treatment

## Abstract

COVID-19 outbreak has put the whole world in an unprecedented difficult situation bringing life around the world to a frightening halt and claiming thousands of lives. Due to COVID-19’s spread in 212 countries and territories and increasing numbers of infected cases and death tolls mounting to 5,212,172 and 334,915 (as of May 22 2020), it remains a real threat to the public health system. This paper renders a response to combat the virus through Artificial Intelligence (AI). Some Deep Learning (DL) methods have been illustrated to reach this goal, including Generative Adversarial Networks (GANs), Extreme Learning Machine (ELM), and Long/Short Term Memory (LSTM). It delineates an integrated bioinformatics approach in which different aspects of information from a continuum of structured and unstructured data sources are put together to form the user-friendly platforms for physicians and researchers. The main advantage of these AI-based platforms is to accelerate the process of diagnosis and treatment of the COVID-19 disease. The most recent related publications and medical reports were investigated with the purpose of choosing inputs and targets of the network that could facilitate reaching a reliable Artificial Neural Network-based tool for challenges associated with COVID-19. Furthermore, there are some specific inputs for each platform, including various forms of the data, such as clinical data and medical imaging which can improve the performance of the introduced approaches toward the best responses in practical applications.

## I. Introduction

The novel Coronavirus designated SARS-CoV-2 appeared in December 2019 to initiate a pandemic of respiratory illness known as COVID-19 which proved itself as a tricky illness that can emerge in various forms and levels of severity ranging from mild to severe with the risk of organ failure and death. From mild, self-limiting respiratory tract illness to severe progressive pneumonia, multiorgan failure, and death [1]–[4]. With the progress of the pandemic and rising number of the confirmed cases and patients who experience severe respiratory failure and cardiovascular complications, there are solid reasons to be tremendously concerned about the consequences of this viral infection [5]. Determining appropriate approaches to reach solutions for the COVID-19 related problems have received a great deal of attention. However, another huge problem that researchers and decision-makers have to deal with is the ever-increasing volume of the date, known as big data, that challenges them in the process of fighting against the virus. This justifies how and to what extent Artificial Intelligence (AI) could be crucial in developing and upgrading health care systems on a global scale [6]. AI has been recently attracted increasing research efforts towards solving the complex issues in a number of fields, including engineering [7]–[9], medicine [10]–[13], economy [14], and psychology [15]. Hence, a critical situation like this necessitates mobilization and saving medical, logistic and human resources and AI can not only facilitate that but can save time in a period when even one hour of the time save could end in saving lives in all locations where Coronavirus is claiming lives. With the recent popularity of AI application in clinical contexts, it can play an important role in reducing the number of undesired deletions as well as improving the productivity and efficiency in studies where large samples are involved [16], and higher degrees of accuracy in prediction and diagnosis are intended [17]. Utilizing big data can also facilitate viral activity modeling studies in any country. The analyses of results enable health care policymakers to prepare their country against the outbreak of the disease and make well-informed decisions [18]. Nevertheless, while treatment strategies, crisis management, optimization and improvement diagnosis methods, such as medical imaging and image processing techniques could take benefit from AI which is potentially capable of helping medical methods, it has not been desirably employed and well-appropriated to serve health-care systems in their fights against COVID-19. For instance, one area that can take special advantage of AI’s useful input is image-based medical diagnosis through which fast and accurate diagnosis of COVID-19 can take place and save lives [19]. Appropriating AI techniques to deal with COVID-19 related issues can fill the void between AI-based methods and medical approaches and treatments. AI specialists’ use of AI platforms can help in making connections between various parameters and speed up the processes to obtain optimum results.

In this paper, our team relies on the findings of the most recent research focusing on COVID-19 and its various challenges to generalize and suggest a variety of strategies relevant but not limited to high-risk groups, epidemiology, radiology and etc. As the paper unfolds, it explores and discusses the potentials of AI approaches to overcome COVID-19 related challenges in section 2. Section 3 of the paper includes a presentation of ANN-based strategies that can be employed for big data analysis. Section 4 presents the discussion, and Section 5 o?ers the conclusion.

## II. Artificial Intelligence and COVID-19

The present section focuses on the introduction of some applicable AI-based strategies that can support existing standard methods of dealing with COVID-19 in health care systems around the world. With the aim of foregrounding the enhanced effectiveness of these strategies and techniques, their formation has been informed by and based on the most recent AI-related published medical updates as well as the latest updates on COVID-19. Therefore, this section presents ideas that can enhance and speed up ANN-based methods obtaining process to improve treatment methods and health management as well as recognition and diagnosis. However, the optimal effectiveness of AI tools during COVID-19 pandemic depends on the extent of human input and collaboration in different roles humans play. The knowledge of capabilities and limitations of AI, however, stays with data scientists who play an important role simply because they are the ones who code AI systems [19].

**FIGURE 1.**
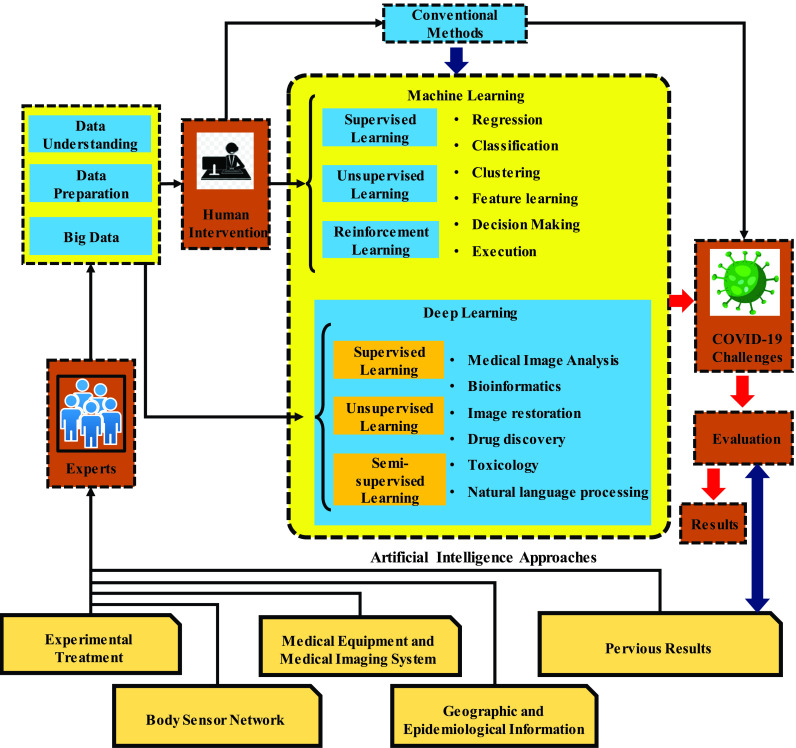
The process of application of AI-based methods to conquer challenges associated with COVID-19.

Different steps in the application of AI-based methods employed to overcome COVID-19 challenges are presented in the flowchart shown in Fig. 1. The first step is the preparation of the data which are necessary for data mining during data understanding, data preparation and big data. The data under discussion here consist of medical information, such as clinical reports, records, images and other various forms of information that can be transformed into data that can be understood by a machine. Objectives of data understanding include understanding data attributes and identifying main characteristics such as data volume and the total number of variables to summarize the data. Before processing and analysis comes data preparation that is the process through which raw data are refined and converted. In other words, it is a process in which data are reformatted, corrected and combined to enriched data. Collecting, analyzing and leveraging the data such as consumer, patient, physical, and clinical data ends in big data. It is at this stage that human intervention, as a part of machine learning methods, takes place and experts investigate and analyze the data to extract the data with finest structures, patterns and features.

Humans’ contribution at this stage is important because their knowledge and potentials are not available to an ML solution that unlike humans is able to deal with huge data sets far beyond the extent that humans could handle or observe in a simultaneous manner. Moreover, Deep Learning (DL) methods could be employed in cases where enormous or complex data processing challenge ML or traditional means of data processing. DL methods, as Fig. 1 demonstrates, are not dependent on human intervention. As a subset of machine learning, DL consists of numerous layers of algorithms that provide a different interpretation of the data it feeds on. However, DL is mainly different from ML because it presents data in the system in a different manner. Whereas DL networks work by layers of Artificial Neural Networks (ANN), ML algorithms are usually dependent on structured data. Unlike supervised learning which is the task of learning a function mapping an input to an output on the basis of example input-output pairs, unsupervised learning is marked by minimum human supervision and could be described as a sort of machine learning in search of undetected patterns in a data set where no prior labels exist. In conventional medicine, alternatively called as allopathic medicine, biomedicine, mainstream medicine, orthodox medicine and Western medicine, medical doctors and other professional health care providers such as nurses, therapists, and pharmacists use drugs, surgery or radiation to treat illnesses and eliminate symptoms.

AI could be extensively applied for COVID-19; however, we aim at finding the best possible solutions COVID-19 related issues that have put the biggest challenges ahead of health care systems. Accordingly, these solutions have been categorized into 3 parts, including high-risk groups, outbreak and control, recognizing and diagnosis.

**FIGURE 2.**
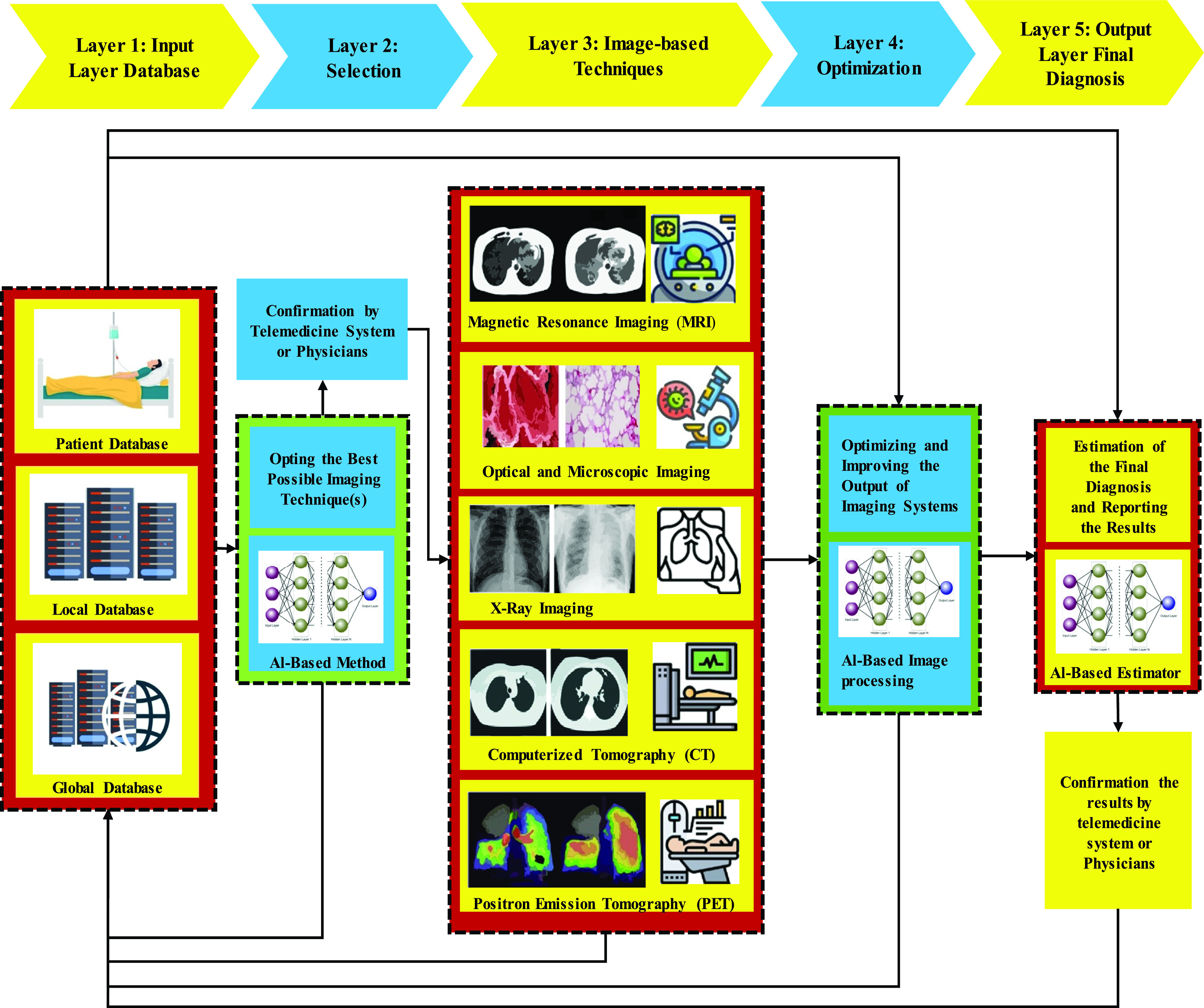
Application of AI-based methods in classification, analysis and improvement of the medical imaging approaches.

Fig. 2 is a flowchart that shows various applications of ANNs in diagnosis and tracing the symptoms in 5 layers. Although the process has been specifically designed for COVID-19 related problems, it has the potential for use in other medical imaging analyses. The input layer as the initial layer is related to the database and is designed for database access. A high-speed channel is used to couple this layer with the main (front-end) computer (s). While the database server is loosely coupled through the network, the database machine is tightly coupled to the main CPU. Taking advantage of a good number of microprocessors with database software database machines can send huge packets of data to the mainframe. The next layer, selection layer, is designed by an intelligent ANN-based selector and has the task of adopting the best possible imaging techniques in the light of past experiences of the system. If physicians confirm the decisions made by this layer, the recommended techniques in the third layer take the required images. Consequently, one or several imaging techniques may be suggested according to the previously obtained results. For each patient, Magnetic Resonance Imaging (MRI), Computed Tomography Scan (CT Scan), positron emission tomography (PET), Optical and Digital Microscopic Imaging Techniques and applications in Pathology and X-Ray imaging are the techniques that may be used in the process. The conventional optical microscope has come to be the dominant tool in pathological examinations. PET scan that, in some cases, detect disease before it can be detected by other imaging tests, is a valuable imaging test to determine the extent and quality of body tissues and organs’ functions [20]–[22]. In the PET scan, a radioactive drug (tracer) is utilized to investigate this functionality [23].

The fourth layer is dedicated to the optimization and improvement of the images. To realize a classification network that facilitates discrimination between COVID-19 and Influenza-A viral pneumonia, a DL technology was used for network structure, and the classical ResNet was used to extract features [24]. The fifth layer is reserved for ultimate diagnosis based on the system’s saved information and is a layer in which learning algorithms should be done by an ANN method. DL technologies, such as a convolutional neural network (CNN), are supposed to be the right option for achieving these goals. The reason is that this type of network is significantly capable of nonlinear modeling and has extensive use in medical image processing and diagnosis process [25]–[28].

## III. The Possible Platform to Accelerate Conventional Methods

Finding solutions for high-risk groups who face COVID-19 is the main concern of the present paper. Since reaching the best possible results is the main objective, we will try to demonstrate ways through which ANN-based methods could be used as complementary to the conventional ones. As [29] suggested it is necessary to keep patients involved COVID-19 registry that highlights clinical variables and cardiovascular complications because it facilitates the identification of the pattern of cardiovascular complications, furthers developing a risk model for cardiac complications, and assists with identification and/or prediction of the response to different types of treatment modalities.

**FIGURE 3.**
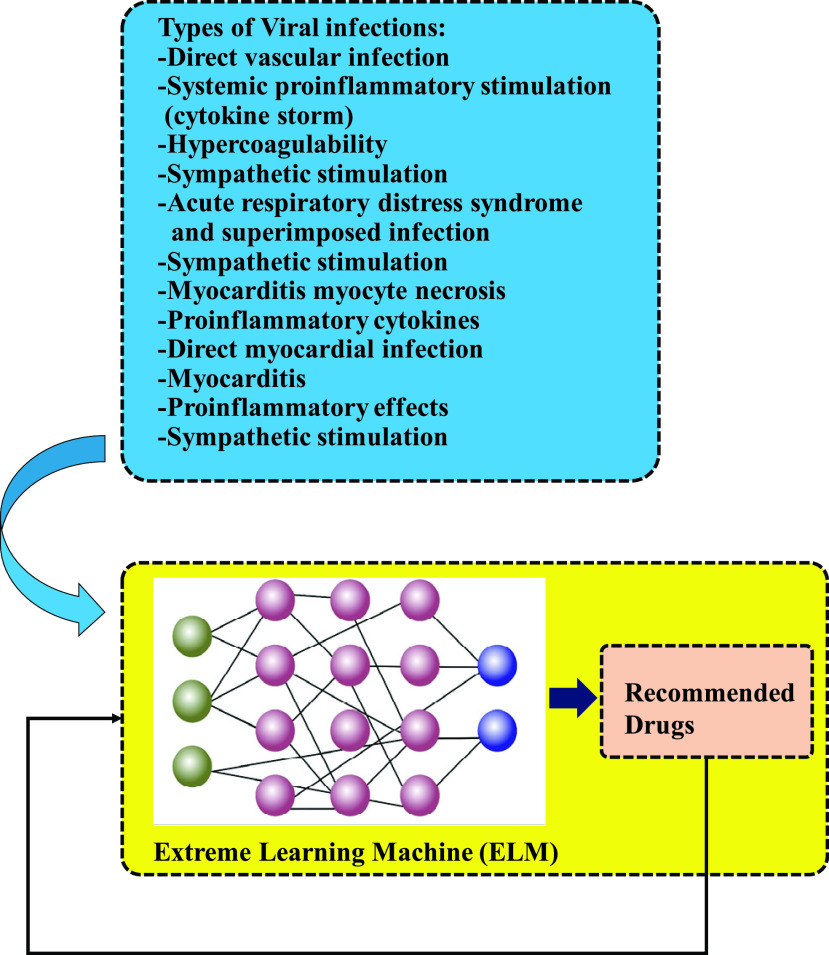
An ELM model to predict suitable drugs based on the performed studied in [29].

Fig. 3 presents an Extreme Learning Machine (ELM) model that relies on the performed studies in [29] to predict suitable drugs based on individuals who are involved with such cardiovascular complications. ELM ANN can use previous examples applied to the model to predict desired outputs. This means that training the supervised model happens through the application of the real data in the network. Therefore, considering various forms of viral infection for previous cases, ELM can suggest the best possible drugs for cardiac complications.

In comparison with conventional feedforward network learning algorithms like back-propagation (BP) algorithm, learning speed in ELM is a greatly faster and obtains better generalization performance [30]. Nevertheless, on many occasions, conventional tuning-based algorithms require a lot less hidden neurons than ELM [31]. There are several other studies that have previously scrutinized ELM with fixed network architectures [30], [32]–[34]. Following the training process, new data can be predicted through a test or verification procedure. As [29] suggested, the Coronavirus may cause vascular inflammation, myocarditis, and cardiac arrhythmias. The suggested model depends on the data that [29] presents to predict the ways that cardiovascular system is affected by the Coronavirus. Therefore, the suggested model is capable of reducing the risk of possible cardiovascular complications. Moreover, it realizes the prediction of response to different treatment modalities because it can predict the pattern of cardiovascular complications. Hence, considering their properties and multiple advantages, ELMs are recommended for such problems.

Another complication that COVID-19 causes in the elderly is heart failure, which requires heart failure specialists stay on guard and design a structured approach to these type of patients and include them in developing algorithms for the care of these patients in early stages until the time when definite universal COVID-19 examinations or clinical trials of antivirals are in place, and deeper understanding of final stages of the disease is realized [35]. Excessive use of fluid and drugs, such as NSAIDs that may change the balance of salt and water in elderly patients, should be avoided. Reference [35] and biomarkers, especially in high-risk elderly patients with underlying structural cardiac disease should be used with care and caution. As such, defining and managing advanced heart failure in the phase of hyper inflammation are important issues for heart specialists [35].

**FIGURE 4.**
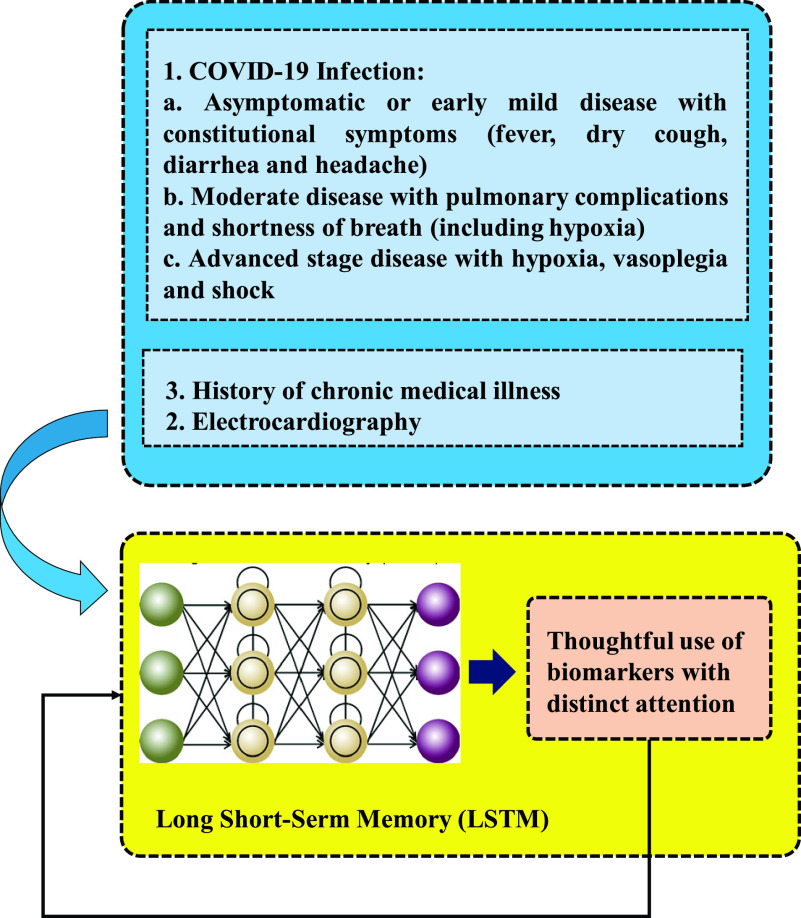
Classifying the best treatment method with high precision through LSTM ANN a developed method inspired by [35].

Fig. 4 shows a model that uses Long/Short Term Memory (LSTM) network put forward in [35]. This model relies on appropriately considered inputs to predict the best treatment as precisely as possible. Being capable of maintaining long memory, LSTM networks are very advantageous for learning sequences with longer-term patterns of unknown length [36].

In addition to electrocardiography and history of chronic medical illness which can help the model training process Mild, moderate and advanced phase of COVID-19 infection can be considered as inputs. Employing multiplicative gates that administer continuous error flow through the internal states of ‘memory cells’ which are special units [36]. LSTM neural networks [37] solve the problem of disappearing gradient in Recurrent Neural Networks (RNNs) Hochreiter and Schmidhuber who were the first to introduce this [37] were followed by others who refined and popularized it [38]. LSTM NN has been popular and increasingly used in robot control, speed recognition, handwriting recognition, human action recognition, etc. over the past ten years [39], and it has worked perfectly in speech recognition [40] and text classification [41], [42]. Reference [43] shows fault prediction to be the main subject in nonlinear systems [44].

**FIGURE 5.**
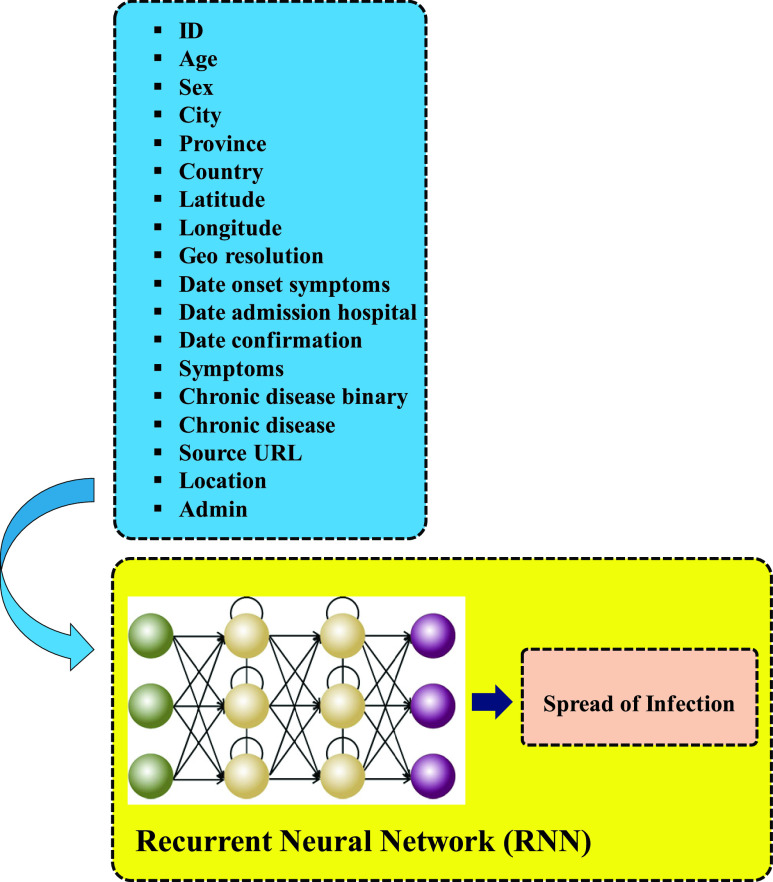
Prediction of spreading the infection by Recurrent Neural Network (GRURNN, Clockwork RNN or CW-RNN)) which is a developed approach based on [45].

ANN-based methods are alternative ways of predicting COVID-19 outbreak. According to [45], a description of the fields in the database is shown here and can be reached via a data dictionary on Github [45] (https://github.com/beoutbreakprepared/nCoV2019/covid19): References to specific settlements along with references to areas that were administrative units have been two ways to collect geographical information. The real-time epidemiological data in [45], have been put together in an organized manner to predict the infection spread. Fig. 5 illustrates how a DL approach, which is powered by RNN can predict the spreading of infection associated with COVID-19 through clinical and geographical big data. Depending on geographical and clinical data, variations of RNNs can be utilized to predict the spread of infection. However, it seems that the best structure to realize the predictions are LSTM network [37], Gated Recurrent Unit RNN (GRURNN) [46], and Clockwork RNN (CW-RNN) [47]. The RNN, as alternatively called Auto Associative or Feedback Network, falls in the category of ANNs in which a directed cycle is made through connections between units [48]. Being a widely appreciated DL family, RNNs have succeeded to present promising results in a lot of machine learning and computer vision tasks [49]. One important task to use this model, however, is the quantification of qualitative inputs such as country and location. Updating the model is possible because of the real-time data by RNN with real-time learning capability. Utilization of the proposed ANN model provides the opportunity of proposing the epidemiological model of the virus in different locations. The main objective of the proposed structure is to improve the accuracy and speed of recognition and classification of the issues caused by the virus by utilizing DL-based methods.

Although screening, diagnosis, and progress assessment of COVID-19 have been effectively performed through reliance on radiological examinations, including CT and digital photography (DR) [50], [51], there has been not much prior experience that could come to help radiologists and technologists to deal with COVID-19 patients. In areas hit by the epidemy, negative RT-PCR but positive CT features are significant signs of COVID-19 and can highlight the importance of rapid detection of the infection that gives the community as well as clinicians a better chance to bring the viral spread under control [52]. While radiological examinations such as computed tomography CT has been demonstrated as effective methods for screening and diagnosis, there is evidence that considerable numbers of radiologists and technologists have been infected while serving COVID-19 patients [50]. Lung CT scans of pneumonia caused by COVID19 picture bilateral, subpleural, groundglass opacities with air bronchograms, illdefined margins, and a slight predominance in the right lower lobe [53]. The image classification model facilitates discrimination of different infections in terms of their appearance and structure. To learn the approximate location information of the patch on the pulmonary image, the model uses relative distance-from-edge as an extra weight [24]. Although the cumbersome task of obtaining a large number of medical images for machine learning applications is possible, specialized and professional reading of diagnostic imaging report that could adroitly address context, syntax, structure, and specific terminologies needed to interpret the imaging is solely left with radiologists who could extract diagnostic information from images and make them available as structured labels for the use of the machine learning model training [54].

**FIGURE 6.**
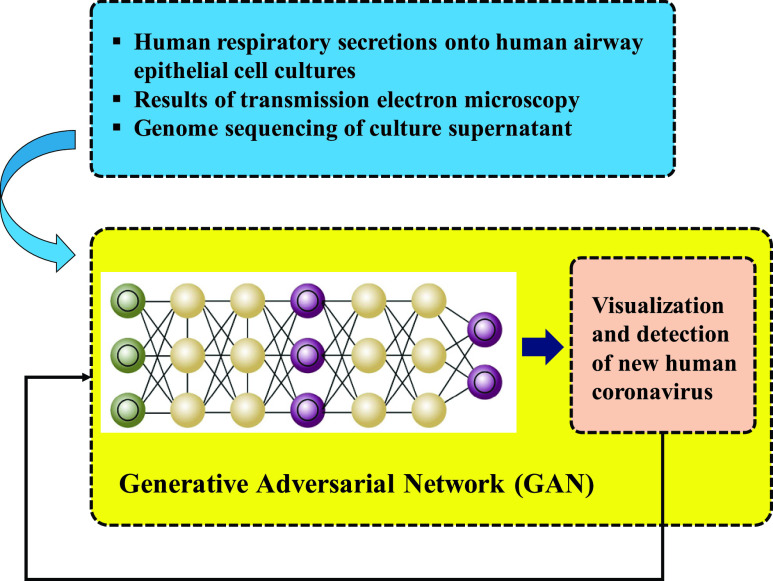
Application of Generative Adversarial Network (GAN) for visualization and detection of new human Coronavirus based on the results of [55].

**FIGURE 7.**
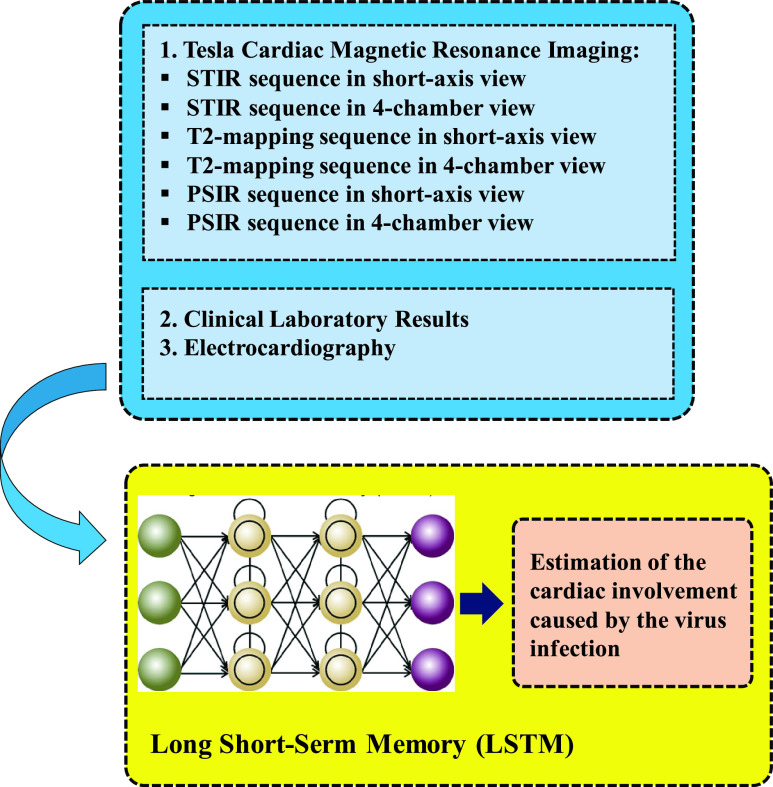
Estimation of cardiac involvement caused by the virus infection extracted from The features from Tesla cardiac magnetic resonance imaging and the information given in [64].

The first case of this part discussed the process of visualization and detection of new human Coronavirus. However, a recent study has shown that initial propagation of human respiratory secretions onto human airway epithelial cell cultures along with transmission electron microscopy and whole-genome sequencing of culture supernatant can be used to visualize and detect new human Coronavirus that has the possibility of remaining unidentified by traditional approaches [55]. As [55] demonstrates infection caused by COVID-19 can damage human airway epithelial cells. It is also demonstrated that visualizing and detecting new human Coronavirus can be done through using the effects of the human respiratory secretions on the human airway along with the results of transmission electron microscopy, and genome sequencing of culture supernatant. Fig. 6 depicts the proposed neural network model and the Generative Adversarial Network (GAN). To analyze electron microscopy images, feature extraction technique can be adopted. GANs are a special type of neural network model in which two networks are trained at the same time while one is focused on generating images, and the other performs discriminating [56]. GANs [57] can solve these problems through effective modelling of the latent distribution of the training data. GANs have successfully been applied to image-to-image translation [58], segmentation [59] and many other subfields of medical image computing [60]. Because of its usefulness in counteracting domain shift, and effectiveness in generating new image samples, the adversarial training scheme has recently attracted a lot of attention. This model has achieved state-of-the-art performance in a lot of tasks, namely text-to-image synthesis [61], super-resolution [62], and image-to-image translation [63]. Those are related to generating images. Another problem to be solved by ANN-based approaches is estimating the extent of cardiac involvement. Reference [64] argues that COVID-19 virus is a major cause of myocarditis. Reference [64] has studied cardiac involvement as a COVID-19 infection capable of causing severe acute respiratory syndrome to conclude that the recognition of acute myocarditis’s association with COVID-19 by the scientific community can be beneficial in monitoring affected patients in a strict manner and could help public health officials in coming to a better understanding of such life-threatening complications. Accordingly, relying on the findings and proposals of [64], an LSTM network is put forward for the estimation of COVID-19 related cardiac involvement. Considering that in feedforward neural networks signals are allowed to merely move in one direction travelling forward from the input to the output. we prefer RNNs because they allow signals to travel both ways introducing loops in the network allowing internal connections among hidden units [65]. Contrary to feedforward neural network, an RNN processes the sequential inputs through a recurrent hidden state in which activation at each step is dependent on the previous one; hence, the ability of the network to exhibit dynamic temporal behavior [49]. Fig. 7 lists the features from Tesla cardiac magnetic resonance imaging that can be utilized for model training.

Also, an AI-based model exists to estimate the behavior of Remdesivir as well as some clinical parameters. As noted in [66], suggest compared to patients with high viral replication and systemic virus dissemination, patients with a viral load decrease in the upper respiratory tract may need various therapeutic approaches depending on viral kinetics monitoring, may be required. However, due to the small number of patients in this case data analysis be done cautiously. Reference [64] has studied clinical and biological data of five COVID-19 patients. To estimate the behavior of Remdesivir, antiviral medication for post-infection treatment for COVID-19, in treatments of the patients as well as hospital stay, ICU stays and symptomatic period, clinical data of these patients including chronic medical illness or history of chronic medical illness, symptoms, age and gender and tests results on hospital admission are utilized. Nevertheless, the numbers of patients were not sufficient for ELM network. ELM is exactly a least-square based learning algorithm for “generalized” single hidden layer feedforward networks (SLFNs), is useful for estimating regression problem or classifying tasks [67].

**FIGURE 8.**
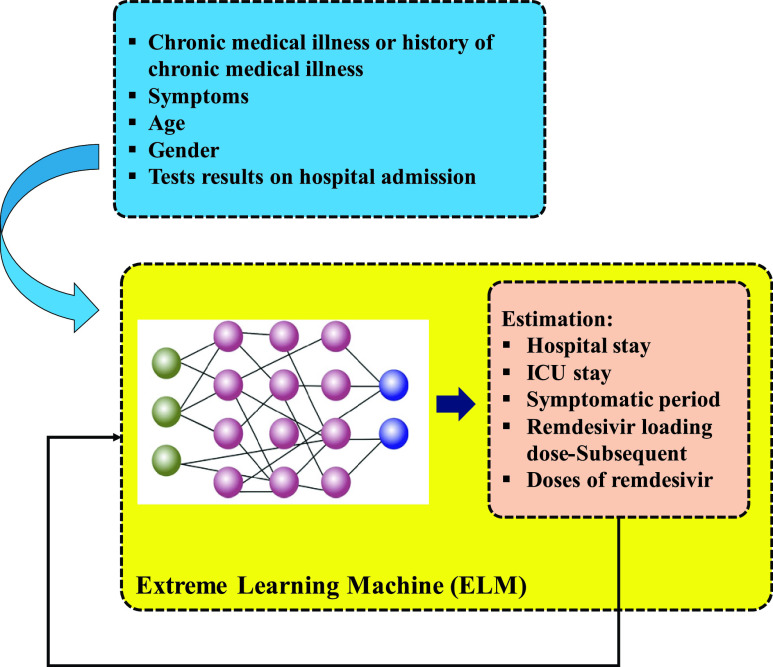
Estimation of Remdesivir drug behavior on the patient’s treatments, hospital stay, ICU stay and symptomatic period using ELM and the ideas of [66].

While input weights (linking the input layer to the hidden layer) and hidden biases in ELM are selected in an arbitrary manner, the output weights (linking the hidden layer to the output layer) are determined in an analytic manner and through the use of Moore–Penrose (MP) generalized inverse [31]. Therefore, ELM technique can be used to train the suggested model. The proposed mentioned ELM model is depicted in Fig. 8.

We propose a model equipped by GAN for viral gastrointestinal infection probability estimation in the last part of the diagnosis system. In [68], evidence for gastrointestinal infection of SARS-CoV-2 and the possibility of faecal-oral transmission route is provided. The spread of the virus from infected to uninfected cells makes viral-specific target cells or organs the main role player in determining the viral transmission routes. The first step of viral infection is the receptor-mediated viral entry into the receiving cell [68]. Besides, ACE2, which is rarely expressed in the oesophagal epithelium, abundantly distributed in cilia of glandular epithelia [68].

However, even after negative conversion of the viral RNA in respiratory tract over 20% of SARS-CoV-2 patients show positive viral RNA in feces which is an indication of viral gastrointestinal infection and the possibility of faecal-oral transmission that can still take place after viral clearance in the respiratory tract [68].

Therefore, routine rRT-PCR testing for SARS-CoV-2 from feces is highly recommended in the case of SARS-CoV-2 patients. Besides, in case rRT-PCR testing demonstrated positive feces test, transmission-Based precautions for hospitalized SARS-CoV-2 patients should be in place [68]. Reference [68] studies the gastrointestinal infection caused by COVID-19. COVID-19-related gastrointestinal infection in this study is evidenced by a collection of images of histological and immunofluorescent staining of rectum, duodenum, stomach and oesophagus. These fluorescent staining images are the output of laser scanning confocal microscopy.

**FIGURE 9.**
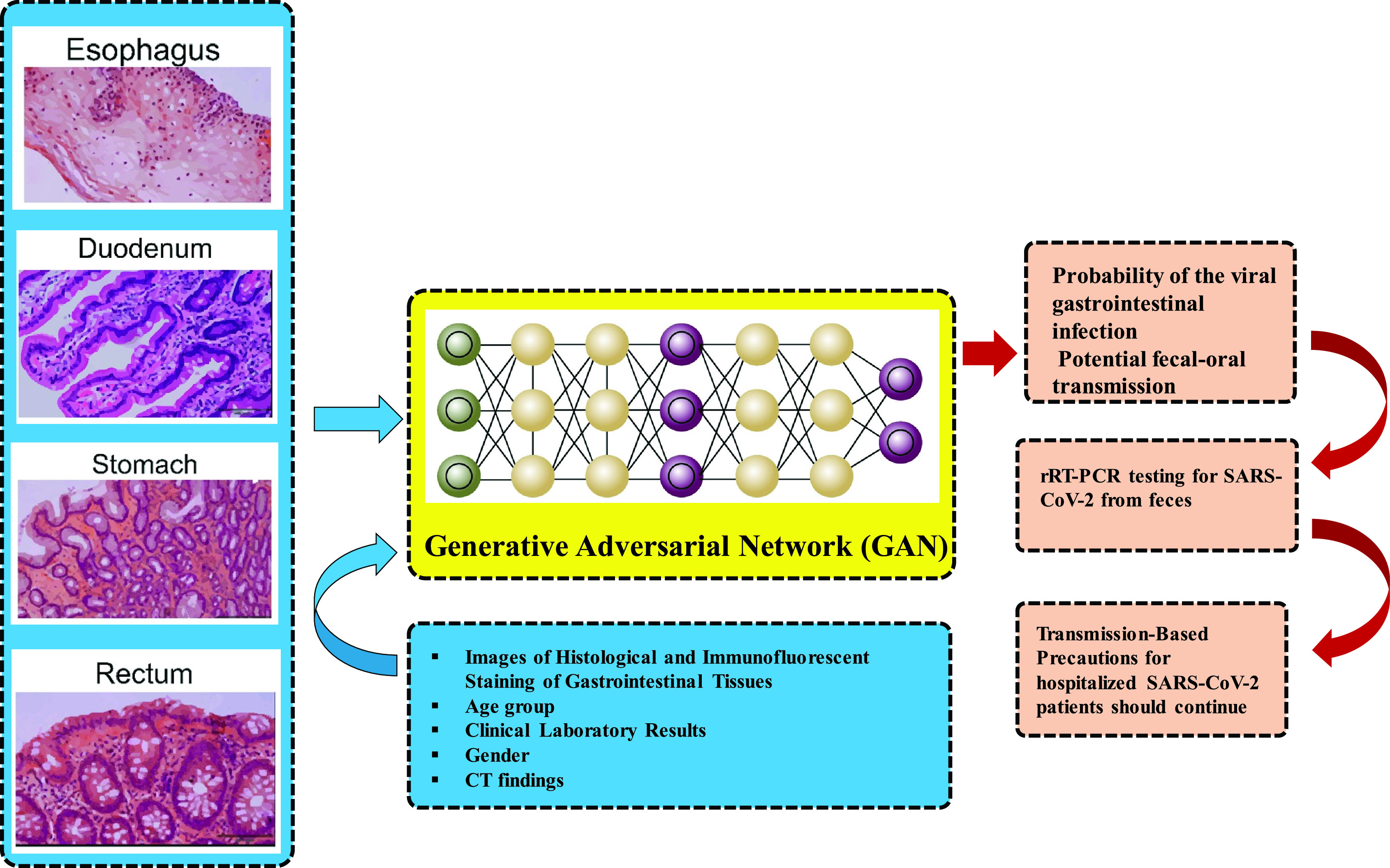
The process of viral gastrointestinal infection probability estimation using a combination of GAN and rRT-PCR testing for SARS-CoV-2 from feces to determine the transmission-based precautions for hospitalized SARS-CoV-2 inspired by [68].

A GAN network to predict viral gastrointestinal infection probability can be done through the extraction of the feature from these images to help patients in the process of their treatment. Fig. 9 presents this model a decision to continue or discontinue transmission-based precautions for hospitalized SARS-CoV-2 patients is dependent on rRT-PCR testing for SARS-CoV-2. The GANs generative process, which projects a standard distribution to complex high-dimensional real-world data distribution stands higher when compared to most discriminative tasks (e.g., classification and clustering) [69]. In addition to image generation tasks, GANs have been introduced to tasks, such as video generation, visual tracking [70], domain adaption [71], hashing coding [72], and feature learning [73].

GANs are of two different users in medical imaging [56]. With their focus on the generative aspect, they facilitate exploration and discovery of the underlying structure of training data and help with learning to generate new images. With their focus on the discriminative aspect, where the discriminator D can be regarded as a learned prior for normal images they can be used as a regularizer or detector when presented with abnormal images [56].

Early screening of COVID-19 patients seems to be effectively managed through DL models demonstrated in this study that can be an effectively helpful supplementary diagnostic method for clinical doctors in close contact with patients [74].

## IV. Discussion

Focusing on the possibility of the ANN application for analyzing COVID-19-related infection problems, such as high-risk patients, control of the outbreak, recognizing and radiology, we used RNN, LSTM, GAN and ELM to suggest several AI-based methods. Advanced machine learning algorithms can integrate and analyze large-scale data related to COVID-19 patients to facilitate a deeper understanding of viral spread pattern, improve the speed and accuracy of diagnosis, develop fresh, effective therapeutic approaches, and even identify individuals who, depending on their genetic and physiological features, are most susceptible to the disease [75]. Despite much praise that such data has received because of its role in improving efficiency, productivity and processes in different sectors, it has been criticized for its small number of users who collect, store, manage the data and have access to them [76]. However, as Heyman maintains AI makes it possible to tell when wrong things are happening, or actions are to be taken regarding COVID-19 because it monitors and collects data coming from social media, newsfeeds, and airliner ticketing systems [77].

A large bulk of various information coming from the most recent advancement and publications in the relevant case can be covered by the suggested methods. Nevertheless, while a variety of inputs exist, clinical data remains as the input shared by almost all the techniques. When it comes to groups that are defined as high risk, overviewing COVID-19 patients’ clinical characteristics throughout pregnancy or disease period is particularly important. The model proposed here is mainly focused on patients with heart failure during the hyper-inflammation phase of this illness and individuals for whom systematic recordings of clinical variables and cardiovascular complications exist. These ideas, however, yield themselves to be extended to other high-risk patients because there are similarities between the structure of ML or DL techniques in complex data estimation and prediction. ELM algorithm is suggested for predicting suitable drugs because it is highly advantageous in problem-solving, but the gradient-based learning algorithms like back-propagation are good to feedforward neural networks with more than one hidden layers. In the case of SLFNs, the present form of the ELM algorithm is valid.

We proposed an LSTM equipped model for the second case, which is the classification of the best treatment method. LSTM networks seem to be good options for classification, process, and prediction according to time series data because lags of unknown duration may take place between major events in a time series. Exploding and vanishing gradient problems that may appear in training traditional RNNs can be effectively dealt with by LSTMs which is proved to be a working tool in cases where sequences exist because in such cases the meaning of a word is dependent on the previous word. Predicting the epidemiology and outbreak by AI was another subject discussed in this paper. The model that we suggested here is based on RNN with a comprehensive set of inputs that can be completed by the database presented in [45]. RNN can be considered a class of ANNs is in which a directed graph along a temporal sequence is formed by connections between nodes making the exhibition of temporal dynamic behavior possible. RNNs’ prediction of the future is influenced by their remembering of past events before learning the underlying relationship of the data when trying to reach the hidden layers RNNs run in a loop. Considering that Imaging workflows can inspire advances in machine learning methods capable of assisting radiologists who seek an analysis of complex imaging and text data, we described models that can analyze medical imaging facilitating the completion of a process that recognizes COVID-19-related infections [54]. As for the epidemic area, we explained that COVID-19 could be the case when negative RT-PCR and positive CT are in place. Considering the importance of rapid detection of the viral infection that can significantly help with more effective control of the viral spread, clinical and societal implications of this argument cannot be ignored [52]. Radiological examinations, such as computed tomography CT, were discussed as effective methods to screen and diagnose infection. It was also mentioned that a considerable number of radiologists and technologists have been infected in the process of examining COVID-19 patients [50]. COVID19 pneumonia is mostly seen on lung CT scans as bilateral, subpleural, groundglass opacities with air bronchograms, illdefined margins, and a slight predominance in the right lower lobe [53].

In the first case of recognizing, visualization and detection of new human Coronavirus by a GAN, the inputs of the proposed network consist of the effects of the human respiratory secretions on the human airway, results of transmission electron microscopy, and genome sequencing of culture supernatant.

It is important to emphasize that COVID-19 is notorious for the rapid deterioration of the function of the respiratory system that often happens in the second week of the disease; therefore, the current wellness of the patients cannot be a guarantee that they are not hit by the disease and safety netting advice has to be taken seriously [78]. This highlights the importance of utilizing an effective ANN-based method in visualizing and detecting new human Coronavirus. When a training set is given to this technique, it learns to generate new data while it uses the same statistics as the training set. It is also demonstrated that GANs are useful for semi-supervised learning [79], fully supervised learning [80] and reinforcement learning [81]. While GANs learn to map from a latent space to a data distribution of interest, the discriminative network discriminates candidates that the generator creates from the true data distribution. The second case of recognizing includes an LSTM approach that estimates cardiac involvement caused by the virus infection. LSTM units come with multiple architectures. One common architecture consists of a cell and three “regulators” or information flow gates inside the LSTM unit: an input gate, an output gate and a forget gate. Keeping track of the dependencies between the elements in the input sequence is done by the cell. While controlling the extent of a new value flow into the cell is the responsibility of input gate., the extent to which a value remains in the cell is controlled by the forget gate, and the extent to which the value in the cell is used to compute the output activation of the LSTM unit is controlled by the output gate. It is recommended, however, that in the third case of recognizing, ELM network does the estimation of Remdesivir’s behavior in patient’s treatments, hospital stay, ICU stay and symptomatic period. Generally, the black-box character of neural networks and ELM network are major concerns that put engineers on guard when it comes to application in unsafe automation tasks.

However, there are a variety of techniques available, such as reducing the dependence on random input, to approach this particular issue [82], [83]. In the last case of recognizing a GAN predicts the probability of viral gastrointestinal infection. Candidate generation is done by the generative network, and evaluation of the candidate is completed by the discriminative network [57]. The contest operates in terms of data distributions. While the generative network learns to map from a latent space to a data distribution of interest, the discriminative network discriminates candidates that the generator creates from the true data distribution and hence the benefits of using this characteristic into an approximate viral gastrointestinal infection.

Although the proposed techniques have not been utilized yet to evaluate their effectiveness, there are many medical reports and valid sources of information proven the efficiency and accuracy of these methods in many different kinds of similar diseases. The most important result here is to generalize such strong methods based on the characteristics of COVID-19.

## V. Conclusion

The introduced conceptual structures and platforms in the research field of AI-based techniques, which are suitable for dealing with COVID-19 issues, have been studied in this paper. Different techniques have been developed, incorporating COVID-19’s diagnostic systems, such as RNN, LSTM, GAN, and ELM. The geographical issues, high-risk people, and recognizing and radiology were the main problems with COVID-19 and have been studied and discussed in this work. Also, we showed a mechanism for selecting the appropriate models of estimation and prediction of desired parameters using a number of clinical and non-clinical datasets. Considering these platforms assists AI experts to analyze huge datasets and help physicians train machines, set algorithms or optimize the analyzed data for dealing with the virus with more speed and accuracy. We discussed that they are desirable because of their potential for creating a workspace while AI experts and physicians could work side by side. However, it should be noted while AI speeds up the methods to conquer COVID-19, real experiments should happen because a full understanding of advantages and limitations of AI-based methods for COVID-19 is yet to be achieved, and novel approaches have to be in place for problems of this level of complexity. Succeeding in the combat against COVID-19 toward its eventual demise is highly dependent on building an arsenal of platforms, methods, approaches, and tools that converge to achieve the sought goals and realize saving more lives.
